# Ketogenic Diet and microRNAs Linked to Antioxidant Biochemical Homeostasis

**DOI:** 10.3390/antiox8080269

**Published:** 2019-08-02

**Authors:** Roberto Cannataro, Maria Cristina Caroleo, Alessia Fazio, Chiara La Torre, Pierluigi Plastina, Luca Gallelli, Graziantonio Lauria, Erika Cione

**Affiliations:** 1 GalaScreen Laboratories, Mater Domini Hospital 88100 Catanzaro and Department of Pharmacy, Health and Nutrition Sciences, University of Calabria, 87036 Rende (CS), Italy; 2 Department of Pharmacy, Health and Nutrition Sciences, University of Calabria, Via Pietro Bucci, 87036 Rende (CS), Italy; 3 Clinical Pharmacology and Pharmacovigilance Operative Unit, Department of Health Science, University of Magna Graecia, Mater Domini Hospital Catanzaro, 88100 Catanzaro, Italy

**Keywords:** ketogenic diet, microRNAs signature, catalase

## Abstract

Recently, we demonstrated the capability of the ketogenic diet (KD) to influence the microRNA (miR) expression profile. Here, we report that KD is able to normalize miR expression in obese subjects when compared with lean subjects. By applying two different bioinformatics tools, we found that, amongst the miRs returning to comparable levels in lean subjects, four of them are linked to antioxidant biochemical pathways specifically, and the others are linked to both antioxidant and anti-inflammatory biochemical pathways. Of particular interest is the upregulation of hsa-miR-30a-5p, which correlates with the decrease of catalase expression protein in red blood cells.

## 1. Introduction

The progress of the obesity pandemic is still substantially underestimated but is alarming [1]. Obese individuals have a lower quality of life and more risk of developing several clinical problems [2]. Obesity is a chronic status with sub-clinical inflammation which is associated with the abnormal synthesis of cytokine/adipokines, leading to an increase of radical oxygen species (ROS) [3]. Therefore, obesity is not *per se* a disease; rather, it is a status that contributes to the imbalance of anti-inflammatory and oxidative stress biochemical pathways [4]. Opportune antioxidant defenses counteract the action of ROS in different organs and are improved by diverse biomolecules [5,6,7], functional food and diet nutrients [8,9]. It is worthy of note that, recently, hyperglycemic crisis was linked to oxidative stress in diabetic patients [10]. While ketoacidosis is not safe for human health, it is well known that the ketogenic diet (KD) is safe. KD is a nutritional regimen in which the amount of carbohydrate is maintained at less than 30 g per day [11]. Although its application initially was a therapeutic regimen for refractory epilepsy, today it is often used to lose weight [12]. KD was proved to possess antioxidant and anti-inflammatory properties as well as to regulate obese subjects in stage 1 of the Edmonton Obesity Staging System (EOSS) and their microRNA (miR) expression profile [11,13,14,15]. The 11 miRNAs analyzed previously in this subject category were normalized with KD when compared to lean subjects. Besides being regulators of the metabolic network in which ROS are always produced, these miRs are also able to counteract inflammatory and oxidative stress [16]. 

## 2. Materials and Methods 

### 2.1. Population

Ethical approval for all human studies was granted in accordance with the Regional Ethics Committee (REC) (#120-18052018). Subjects were also excluded if they showed the presence of hypertension and/or were on medication. The study was considered not to have set up clinical trials and was not registered as such. Written informed consent was obtained from participants which conformed to the standards of ethical practice as outlined in the declaration of Helsinki. The exclusion criteria included diabetes, renal diseases, liver dysfunction, a history of alcohol or drug abuse, and neoplastic diseases in the five years prior to the study.

### 2.2. Immunoblot Analysis and RNAs Extraction

Blood samples were collected, and red blood cells and lysates for catalase (CAT) expression protein were harvested using the antibody (abcam # ab16731). The protein concentration in samples was estimated as described in [17,18,19,20]. Serum plasma was then used for functional genomics assay. Total RNA was extracted from 200 μL of blood serum or plasma by using an miRNeasy Serum/Plasma Kit (Qiagen, Venlo, The Netherlands) in order to lower potential contaminants [15,21] and according to the manufacturer’s instructions. 

### 2.3. NanoString Sample Preparation and Data Analysis

For the n-counter flex of NanoString Technology, 100 ng of RNA/miR was used as input. miRs were then hybridized with an nCounter Human-V3 miRNA Expression Assay CodeSet overnight at 65 °C and as previously described in [22,23]. In order to obtain robust results, the coefficient of variation (CV), expressing the ratio of the standard deviation to the mean and expressed as a percentage, was chosen as 30%. The miRs known to be linked to blood hemolysis were excluded from the analysis [24,25].

### 2.4. In Silico Prediction of hsa-miR Target Genes

In order to identify genes as targets of hsa-miRs from the array analysis, we performed in silico analysis. The in silico identification of the target genes was performed using miRTargetLink Human (https://ccb-web.cs.uni-saarland.de/mirtargetlink/) and DIANA Tools (http://diana.imis.athena-innovation.gr/DianaTools/index.php) databases. This latter database was used to check which miRNA target genes were already validated experimentally.

### 2.5. Statistical Analysis 

Prism GraphPad Prism version 5.0 for Windows (GraphPad Software, San Diego, CA, USA) was used to plot the results. Differences within and between groups were evaluated by the *t*-test and one-way ANOVA followed by a multi-comparison Bonferroni test (* *p* < 0.05).

## 3. Results 

### 3.1. Characteristics of Subjects 

A total of 43 subjects, divided into categories of obese, lean and on a ketogenic diet (KD), were selected, with numbers for obese subjects of *n* = 14, lean of *n* = 17 and KD of *n* = 12. The subjects’ characteristics are described in Table 1.

### 3.2. Comparison of Obese, Lean and KD Array Profiles

**Figure 1:**
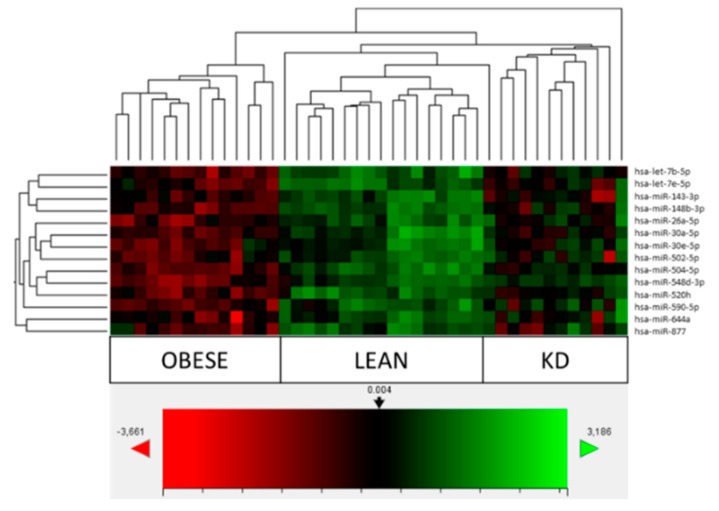
Heatmap and hierarchical clustering of obese (*n* = 14), lean (*n* = 17) and ketogenic diet (KD) subjects (*n* = 12) based on the most differentially expressed microRNAs. The color and the intensity of the boxes represent changes of gene expression. In the analysis, red represents down-regulated genes and green represents up-regulated genes. Black represents an unchanged expression as evident by the color reference. *n*-Solver software was used.

The heatmap and hierarchical clustering based on the most differentially expressed hsa-miRs are shown in Figure 1, including the signatures of hsa-let-7b-5p, hsa-miR-143-3p, hsa-miR-148b-3p, hsa-miR-26a-5p, hsa-miR-502-5p, hsa-miR-520h, hsa-miR-548d-3p, hsa-miR-590-5p and hsa-miR-644a. In particular, KD, compared to obese subjects, normalized the expression levels of hsa-let-7b-5 (8/12), hsa-miR-143-3p (9/12), hsa-miR-148b-3p (10/12), hsa-miR-590-5p (10/12), hsa-miR-520h (8/12) and hsa-miR-644a (9/12), which were expressed in more than 65% of subjects, while 100% was achieved for hsa-miR-548d-3p (12/12). At least 50% was reached for hsa-miR-26a-5p (6/12), and hsa-miR-502-5p (7/12). No change was seen for hsa-miR-504-5p. The new hsa-miR-let7e-5p (5/12) and hsa-miR-877 (5/12) here identified showed a less than 50% presence, with the exception of hsa-miR-30a-5p (8/12).

### 3.3. In Silico Results 

Two different databases were used for the in-silico analysis. Data were compared with respect to the number of target genes experimentally validated in both databases. The results are reported in Table 2. Although similar results were found for two hsa-miRs (in bold in Table 2), the numbers of validated targets found for others hsa-miRs were consistently different. In DIANA tools, the numbers of validated target genes were higher in respect to miRtagertLink Human; therefore, DIANA tools were used for further bioinformatics analysis.

### 3.4. Validated hsa-miR Interaction and Western Blot Analysis of Catalase

**Figure 2:**
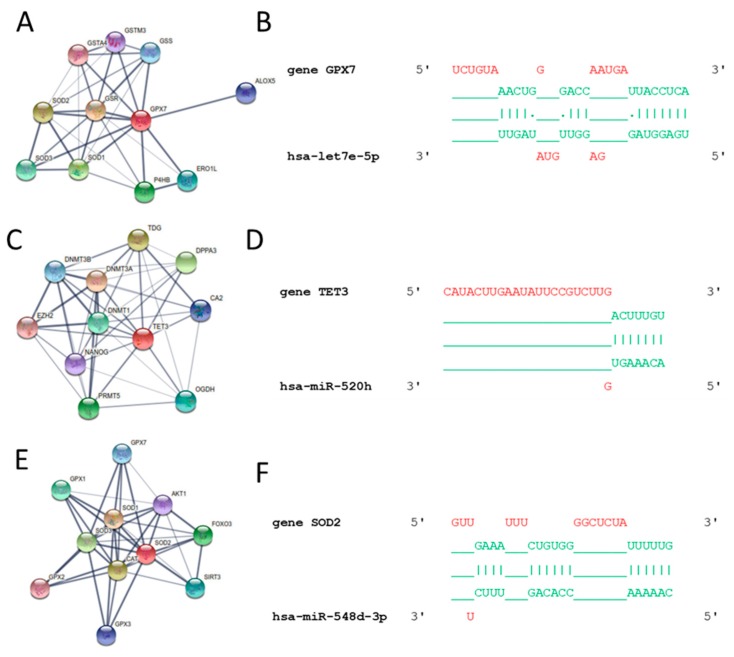
Bioinformatics analysis. String protein interaction of (**A**) GPX7 and (**B**) GPX7 in silico interactions with the 3′UTR region. String protein interaction of (**C**) TET3 and its in silico interactions with the 3′UTR region in (**D**). String protein interaction of (**E**) SOD2 and its in silico interactions with the 3′UTR region in (**F**).

**Figure 3:**
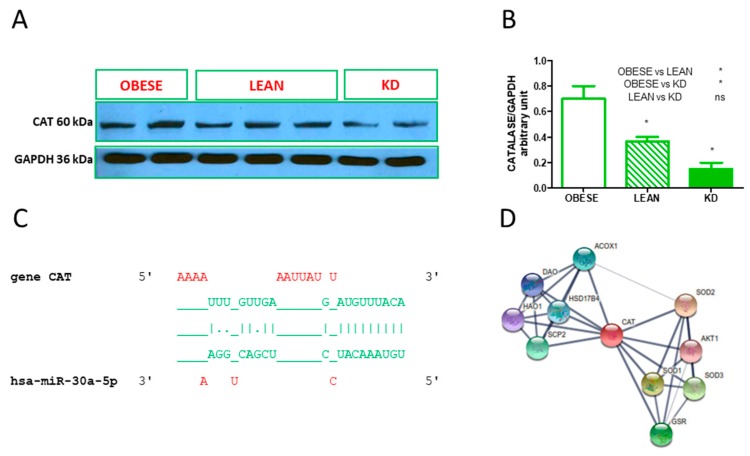
A ketogenic diet (KD) influences catalase gene expression. (**A**) Western blot analysis of catalase (CAT) protein levels in red blood cells from obese, lean and subject in KD. (**B**) Graphical representation of Western blot band intensity, normalized with the loading control GAPDH. (**C**) The CAT gene 3′UTR region interacts with hsa-miR-30a-5p. (**D**) The protein network of CAT enzymes determined by string analysis. Data in panel B represent means ± SD of *n* = 2 for obese, *n* = 3 for lean and *n* = 2 for KD independent tests (* *p* < 0.05).

Predicted and validated target genes were assessed using DIANA Tools. The new hsa-miR-let7e-5p was found to regulate glutathione peroxidase 7 (GPX7), as shown by string analysis (Figure 2A), as well as in silico 3′UTR interaction (Figure 2B). In silico 3′UTR regions of tet methylcytosine dioxygenase 3 (TET3) for hsa-miR-520h (Figure 2D) interaction and 3′UTR regions of superoxide dismutase 2 (SOD2) for hsa-miR-548d-3p (Figure 2F) are shown, as well as string analysis, in Figure 2C,E, respectively. String analysis showed that 10 proteins are able to physically interact with GPX7 (Figure 2B) as well as with TET3 (Figure 2D), SOD2 (Figure 2F) and catalase (CAT) (Figure 3D). This latter protein was monitored through Western blot analysis, as shown in Figure 3A. Densitometric analysis was performed and exhibited a significant decrease of CAT protein levels, as shown in Figure 3B. The hsa-miR-30a-5p was found to target 3′UTR regions of CAT, as shown in Figure 3C. However, other target genes were found to be influenced by KD and linked to antioxidant metabolism and inflammatory-related genes, as shown in Table 3. Abbreviations and gene names are described in Table 4.

## 4. Discussion

The World Health Organization states that obesity has nearly tripled since 1975. In 2016, more than 1.9 billion adults were overweight. Of these, over 650 million were obese, leading to the following report sentence: “*Most of the world’s population live in countries where overweight and obesity kills more people than underweight. Obesity is preventable*” [26]. How can obesity be preventable? One method is represented by KD. The beneficial effects of KD in reducing the body weight and body mass index of obese subjects over both the short (6 weeks) and long (24 weeks) term has already been proven [12,27]. The administration of KD for a relatively long period is safe and can be considerate a nutritional therapy for weight reduction in obese patients [27]. Besides that, to keep KD beneficial, lifestyle change is mandatory and is the harder part of nutritional intervention during KD, with documented improvement of the endurance exercise capacity, better recovery from fatigue and exercise-induced muscle and organ damage prevention in obese subjects. Besides that, the anti-inflammatory action of physical activity was also recently reviewed [28,29]. Nutraceuticals with antioxidant properties were proposed to help in the treatment of obesity, but they are not enough when taken alone [30]. Several scientific approaches to date have tried to describe this disorder by way of genetic or environmental factors [31]. The role of epigenetics in human diseases has been well described relatively recently. Obesity and epigenetics is a consolidated union [32,33,34], and bariatric surgery induces epigenetic change in obese subjects [35]. We recently reported the influence of the ketogenic diet (KD) on the circulating microRNA (miR) expression profile [15]. The 11 miRs controlling the metabolic network identified so far in subjects on KD were almost normalized and closer to lean subjects. Besides that, new miRs targeting identified genes linked to the homeostasis of oxidant–antioxidant pathways were identified. These latter molecules act as epigenetic regulators and have the peculiarity of regulating gene expression targeting the 3′UTR mRNA region [31]. Predicted and validated target genes were assessed using DIANA Tools. The hsa-miR-let7e-5p has a role in pathogen recognition [36]. A low level of hsa-miR-520h was found in the alteration of the placenta, mediated by oxidative stress [37], and hsa-miR-548d-3p was found to be involved in the control of the homeostasis of oxidative stress damage, the metabolic network and survival pathways [38]. Here, we found an in silico interaction of that miR with glutathione peroxidase 7 (GPX7), tet methylcytosine dioxygenase 3 (TET3) and superoxide dismutase 2 (SOD2). GPX7 is a glutathione peroxidase homolog for which the exact biochemistry is not fully understood [39]. TET3, which is aberrantly expressed in acute myeloid leukemia, promotes DNA oxidation [40,41]. The mitochondria-localized manganese superoxide, SOD2, has a dichotomous role and aids in the regulation of several types of cancers [42]. All those proteins directly or indirectly exhibit a physical interaction with CAT. This latter protein was monitored through Western blot analysis and decreased after KD regime. The hsa-miR-30a-5p was found to target the 3′UTR regions of CAT. It is worth noting that the family of hsa-miR-30, to which hsa-miR-30a-5p belongs, is a promising regulator in both development and disease [41]. In the interplay of oxidative stress, pro-oxidants, and antioxidants, this is already known [43]. In particular, the regulation of antioxidant genes such as SOD, CAT, and GPX was studied in mice models kept in KD. The short time-frame of KD did not affect the SOD expression protein while it significantly decreased both GPX and CAT [44].

## 5. Conclusions

Modulating miRs linked to antioxidant and inflammatory states in obese people might be the key to the success, in particular in the long term, of a nutritional program. The reciprocal action of diet and nutrients on anti-oxidant and anti-inflammatory miRs can present tools to predict and follow the success of a nutritional programs.

## Figures and Tables

**Table 1:** Subject characteristics.

Characteristic	Obese (*n* = 14)	Lean (*n* = 17)	KD (*n* = 12)	*p* Value *
Age, y	46.5 ± 10.51	46.83 ±12.32	46.6±11.56	ns
Height, cm	175.1 ± 5.2	171.3 ± 6.6	176.3 ± 3.3	ns
Weight, kg	107.5 ± 3.0	70.8 ± 3.8	96.97 ± 11.2	<0.001
BMI, kg/m^2^	33.9 ± 1.2	22.1 ± 2.5	31.5 ± 1.3	<0.001

Data are presented as mean ± SD. * Using *t* test.

**Table 2:** Bioinformatics tools for in silico analysis.

Number of Target Genes		
	miRTargetLink Human	DIANA Tools
hsa-let-7b-5p	124	312
hsa-let-7e-5p	15	273
hsa-miR-143-3p	32	82
hsa-miR-148b-3p	10	218
hsa-miR-26a-5p	52	391
hsa-miR-30a-5p	119	458
hsa-miR-30e-5p	7	412
hsa-miR-502-5p	3	30
**hsa-miR-504-5p**	**6**	**7**
**hsa-miR-520h**	**5**	**5**
hsa-miR-548d-3p	1	203
hsa-miR-590-5p	2	43
hsa-miR-644a	2	0
hsa-miR-877	0	19

**Table 3:** Antioxidant metabolism and inflammatory-related genes.

Biochemical Pathways and Possible miRs Gene Interaction		
	miRNA	Validated target genes
Glutathione metabolism	hsa-let-7b-5p	GPX7, GSR, RRM2, GGCT
	has-let-7e-5p	GPX7
	hsa-miR-26a-5p	RRM2
Chondroitin sulfate biosynthesis	hsa-let-7b-5p	CHPF2, XYLT2
Arachidonic acid metabolism	hsa-let-7b-5p	CYP2J2, GPX7, LTA4H, PTGS1, PTGS2, PTGES2
	hsa-miR-26a-5p	PTGS1
	hsa-miR-143-3p	PTGS2
Toll like receptor signalling pathway	hsa-let-7b-5p	IFNB1, NFKBIA, MAPK1, MAP2K2, TAB2
	hsa-miR-26a-5p	IFNB1, IL6
	hsa-miR-30e-5p	CAT
	hsa-miR-877-5p	MAPK8
	hsa-miR-148b-3p	PIK3CA, PIK3CG
	hsa-miR-143-3p	AKT1
	hsa-miR-520h	TET3
Natural killer cell mediated cytotoxicity and T Cell, B Cell receptor signalling pathways	hsa-let-7b-5p	IFNB1, NFATC1, NFATC3, NRAS, NFKBIA, PAK1, MAPK1, MAP2K2, PDK1, CD81
	hsa-miR-26a-5p	IFNB1, SHC2, IL6
	hsa-miR-30e-5p	RELA, CAT
	hsa-miR-504-5p	FAS
	hsa-miR-877-5p	NFAT5, NRAS, PIK3CCA
	hsa-miR-143-3p	HRAS, KRAS, AKT1
	hsa-miR-148a-3p	HLA-G, CCL28
	hsa-miR-548d-3p	AKT3, SOD2

**Table 4:** Abbreviations and gene names.

Abbreviation	Gene Name
AKT1	Serine-threonine protein kinase 1
AKT3	Serine-threonine protein kinase 3
CAT	Catalase
CCL28	C-C motif chemokinine 28 precursor
CD81	CD81 antigen target proliferate antibody 1
CHPF2	Chondroitin polymerizing factor 2
CYP2J2	Cytochrome P450 2J2
FAS	FAS cell surface deat receptor
GGCT	Gamma-glutamylcyclotransferase
GPX7	Glutathione peroxidase 7
GSR	Glutathione disulphide reductase
HLA-G	HLA Class I Histocompatibility Antigen, Alpha Chain G
HRAS	Hras protogoncogene GTPase
IFNB1	Interferon beta 1
IL6	Interleukin-6
KRAS	Kras protogoncogene GTPase
LTA4H	Leukotriene-A4 hydrolase
MAP2K2	Mitogen-activated protein kinase 2
MAPK1	Mitogen-activated protein kinase 1
MAPK8	Mitogen-activated protein kinase 8
NFAT5	Nuclear factor of activated T-cells 5
NFATC1	Nuclear factor of activated T cells 1
NFATC3	Nuclear factor of activated T cells 3
NFKBIA	NFKB inhibitor alpha
NRAS	NRAS-proto-oncogene
PAK1	Serine/threonine-protein kinase
PDK1	Phosphoinositide-dependent kinase-1
PIK3CA	Phosphaidylinositol-3-kinase
PIK3CG	Phosphaidylinositol-4,5-Bisphosphatase 3-kinase
PTGES2	Prostaglandin-E synthase 2
PTGS1	Prostaglandin-endoperoxidase synthase 1
PTGS2	Prostaglandin-endoperoxdase synthase 2
PTES2	Prostaglandin-E synthase 2
RELA	RELA-proto-oncogene
RRM2	Ribonucleotide reductase regulatory subunit M2
SHC2	SHC-trasorming protein 2
TAB2	TGF-beta activated kinase 1 binding protein 2
XYLT2	Xylosyltransferase 2
